# Silencing HOXC13 exerts anti-prostate cancer effects by inducing DNA damage and activating cGAS/STING/IRF3 pathway

**DOI:** 10.1186/s12967-023-04743-x

**Published:** 2023-12-06

**Authors:** Maozhang Li, Guangwei Bai, Yi Cen, Qitong Xie, Jiahong Chen, Jia Chen, Qingbiao Chen, Weide Zhong, Xiaobo Zhou

**Affiliations:** 1 School of Medicine, Jinan University, Guangzhou, 510632 Guangdong China; 2 Department of Urology, Huizhou Municipal Central Hospital, Huizhou, 516001 China; 3 Guangdong Provincial Key Laboratory of Molecular Target & Clinical Pharmacology, the NMPA and State Key Laboratory of Respiratory Disease, Guangzhou Medical University, Guangzhou, 511436 People’s Republic of China; 4 Department of Urology, The Second People’s Hospital of Foshan, Affiliated Foshan Hospital of Southern Medical University, Foshan, 528000 China

**Keywords:** Prostate cancer, HOXC13, cGAS/STING/IRF3 pathway, IFN-β, Immune infiltration

## Abstract

**Background:**

Advanced prostate cancer (PCa) will develop into castration-resistant prostate cancer (CRPC) and lead to poor prognosis. As the primary subtype of CRPC, CRPC-AR accounts for the major induction of PCa heterogeneity. CRPC-AR is mainly driven by 25 transcription factors (TFs), which we speculate may be the key factors driving PCa toward CRPC. Therefore, it is necessary to clarify the key regulator and its molecular mechanism mediating PCa progression.

**Methods:**

Firstly, we downloaded transcriptomic data and clinical information from TCGA-PRAD. The characteristic gene cluster was identified by PPI clustering, GO enrichment, co-expression correlation and clinical feature analyses for 25 TFs. Then, the effects of 25 TFs expression on prognosis of PCa patients was analyzed using univariate Cox regression, and the target gene was identified. The expression properties of the target gene in PCa tissues were verified using tissue microarray. Meanwhile, the related mechanistic pathway of the target gene was mined based on its function. Next, the target gene was silenced by small interfering RNAs (siRNAs) for cellular function and mechanistic pathway validation. Finally, CIBERSORT algorithm was used to analyze the infiltration levels of 22 immune cells in PCa patients with low and high expression of target gene, and validated by assaying the expression of related immunomodulatory factor.

**Results:**

We found that HOX family existed independently in 25 TFs, among which HOXC10, HOXC12 and HOXC13 had unique clinical features and the PCa patients with high HOXC13 expression had the worst prognosis. In addition, HOXC13 was highly expressed in tumor tissues and correlated with Gleason score and pathological grade. In vitro experiments demonstrated that silencing HOXC13 inhibited 22RV1 and DU145 cell function by inducing cellular DNA damage and activating cGAS/STING/IRF3 pathway. Immune infiltration analysis revealed that high HOXC13 expression suppressed infiltration of γδ T cells and plasma cells and recruited M2 macrophages. Consistent with these results, silencing HOXC13 up-regulated the transcriptional expression of IFN-β, CCL2, CCL5 and CXCL10.

**Conclusion:**

HOXC13 regulates PCa progression by mediating the DNA damage-induced cGAS/STING/IRF3 pathway and remodels TIME through regulation of the transcription of the immune factors IFN-β, CCL2, CCL5 and CXCL10.

**Supplementary Information:**

The online version contains supplementary material available at 10.1186/s12967-023-04743-x.

## Introduction

Prostate cancer (PCa) is one of the most common malignant tumors of the male genitourinary system and the second leading cause of cancer death in men [1, 2]. PCa has androgen- dependent growth characteristics, androgen deprivation therapy (ADT) is the primary treatment for this type of patients [3]. However, after an average of two years with ADT, most patients progress to castration-resistant prostate cancer (CRPC), implying that ADT is ineffective for PCa and tumors can grow in a hypoandrogenic environment [4]. CRPC has a highly heterogeneous clinical course [5]. Patients with painless disease can survive for years without progression, while aggressive disease can rapidly metastasize and become incurable. Although the incidence of PCa has generally decreased in recent years, a steady increase in the incidence of metastatic CRPC has been observed, which suggests a change in treatment strategy [6]. Therefore, it is necessary to further reveal the formation mechanisms of CRPC heterogeneity, for which tumor subtypes may provide valuable molecular information.

Today, high-throughput sequencing for epigenomics provides potent technical support for studying tumor heterogeneity, revealing the heterogeneity among tumor cells and the complexity of the tumor microenvironment (TME) [7]. In a recent study, researchers using assay for transposase-accessible chromatin sequencing (ATAC-seq) combined with RNA sequencing (RNA-seq) on 22 organoids, 6 patient-derived xenografts and 12 cell lines revealed that CRPC consists of four molecular subtypes, with CRPC-AR as the primary subtype driven by 25 key TFs (AR, FOXA1, PGR, FOXO3, FOXB2, FOXK1, GATA2, FOXO4, HOXA13, HOXB13, FOXJ2, HOXC12, FOXN3, NFYB, FOXP3, FOXL2, NFIC, HOXC13, FOXC2, FOXS1, HSF4, ARID5A, HNF1B, HOXC10 and HNF1A) [8]. These 25 TFs have important regulatory roles in PCa progression, among which FOXA1, FOXK1, GATA2, HOXA13 and FOXC2 have been proven to be oncogenic, while FOXO3, HOXB13, FOXP3 and HNF1B have opposite effects [9–17]. The aberrant functional properties of these TFs in PCa suggest that they are key drivers of TME heterogeneity. TME is a complex environment filled with various cell subtypes, including tumor cells, blood vessels, immune cells and stromal cells, of which tumor immune microenvironment (TIME) is the most important component [18]. Numerous studies have demonstrated that TIME play an important role in the physiological regulation of acquired drug resistance and anti-apoptosis in tumors [19–21]. Therefore, whether these TFs mediate alterations in TIME to affect PCa progression aroused our research interest.

We focused on 25 TFs and analyzed their expression properties and clinical features in PCa by bioinformatics to uncover the key regulator driving PCa progression. The molecular mechanism mediating PCa function was further explored by in vitro experiments. The effects of the target gene on TIME alterations were analyzed by immune infiltration. Our study may provide a novel molecular target for PCa therapy and guide clinical therapeutic decision.

## Materials and methods

### Data collection and processing

RNA-seq expression profiles of 498 prostate adenocarcinoma (PRAD) samples and 52 paracancerous samples were downloaded from The Cancer Genome Atlas (TCGA) database (https://portal.gdc.com), and the corresponding clinical information for TCGA-PRAD cohort was obtained from cBio Cancer Genomics Portal (cBioPortal) database (http://cbioportal.org). For RNA-seq expression profiles, the data type we downloaded was fragments per kilobase per million (FPKM), which was converted to transcripts per million (TPM) expressed as log2 (TPM + 1) for subsequent analysis.

### Gene differential expression analysis

Gene expression levels in normal and tumor tissues were compared according to log2 (TPM + 1). Further, TCGA-PRAD cohort was divided into two groups based on Gleason score (GS, < 8 vs. ≥ 8), tumor stage (T stage, T1–T2 vs. T3–T4), lymph node stage (N stage, N0 vs. N1) and metastasis stage (M stage, M0 vs. M1) to compare the gene expression levels under different clinical features. The statistical difference of two groups was compared by Wilcox test without normal distribution. The analysis method and R package (ggplot2) were implemented by R v4.0.3 software. P < 0.05 was considered statistically significant.

### Protein–protein interaction (PPI) analysis

PPI for multiple proteins was analysed using String database (https://cn.string-db.org/). Active interaction sources for PPI include textmining, experiments, databases, co-expression, neighbourhood, gene fusion and co-occurrence, and the interaction score was set to 0.4 (medium confidence). Further, k-means clustering method was applied to cluster multiple proteins to find characteristic protein clusters. P < 0.05 was considered statistically significant.

### Gene ontology (GO) enrichment analysis

GO terms in biological processes, molecular functions and cellular components were enriched and analysed by calculating strength (enrichment index) and false discovery rate (FDR) based on PPI network counts. FDR < 0.05 was considered statistically significant.

### Co-expression correlation analysis

Based on RNA-seq expression profiles from 498 PRAD samples, Spearman's correlation analysis was used to describe correlation between quantitative variables without normal distribution. Correlation heatmap was displayed by R package (pheatmap) and implemented by R v4.0.3 software. P < 0.05 was considered statistically significant.

### Overall survival (OS) analysis

Based on 498 PRAD samples with survival information, TCGA-PRAD cohort was divided into low expression (n = 249) and high expression (n = 249) groups according to the median expression value of each gene. The effects of gene expression on the prognosis of PRAD patients were analysed using univariate Cox regression and log-rank test. P values and hazard ratio (HR) with 95% confidence interval (CI) were calculated and survival curves were plotted. All the analysis methods and R packages (ggplot2, forestplot and survival) were implemented by R v4.0.3 software. P < 0.05 was considered statistically significant.

### Gene-pathway correlation analysis

The marker genes in the corresponding pathway were collected and analysed by R package (GSVA), and the correlation between target gene and pathway scores was analysed by Spearman's correlation. All the analysis method and R package were implemented by R v4.0.3 software. P < 0.05 was considered statistically significant.

### Tissue microarray information

Tissue microarray (Cat No. = HProA150CS01, n = 150; OUTDO BIOTECH, China), including 50 paracancerous tissue samples and 100 prostate tissue samples from patients with primary PCa, were available with clinicopathologic information. Patients treated with chemotherapy or radiotherapy before the surgery were excluded from this study.

### Immune infiltration analysis

Based on RNA-seq expression profiles of 498 PRAD samples, TCGA-PRAD cohort was divided into low expression (n = 249) and high expression (n = 249) groups according to the median expression value of the target gene. We performed a reliable immunoscoring assessment using the R software package (immunedeconv). CIBERSORT algorithm was used to calculate the enrichment score of each sample for each immune cell to compare the infiltration levels of 22 immune cells in the two groups. All the analysis methods and R packages (gglpot2 and GSVA) were implemented by R v4.0.3 software. P < 0.05 was considered statistically significant.

### Immunohistochemistry (IHC) staining

Tissues were sequentially sectioned (5 μm), dewaxed (65 °C, 2 h), antigen repaired (sodium citrate), blocked (5% BSA), and sealed at room temperature (24 °C, 20 min). Primary antibodies against HOXC13 (1:100, ABclonal, China) was added and incubated overnight at 4 °C. After adding secondary antibody and incubating at room temperature for 1 h, DAB color development, hematoxylin counterstaining, dehydration and sealing were performed successively. The positive cell rate and the degree of staining were scored by scanning imaging. Positive cell rate score: 0–10%, 1 point; 10–50%, 2 points; 50–75%, 3 points; 75–100%, 4 points. Staining degree score: no positive staining, 0 point; canary yellow, 1 point; brownish yellow, 2 points; tan, 3 points. Immune risk score (IRS) is the product of the above two scores.

### Cell line culture

Human PCa cell lines, DU145 and 22RV1, were purchased from BNCC (Beijing, China). Cells were maintained in DMEM (Gibco, USA) with 10% FBS (Gibco, USA) and 100 µg/mL penicillin/streptomycin (Gibco, USA) in a humidifed atmosphere containing 5% CO_2_ at 37 °C.

### siRNAs construction and transfection

siRNAs specific to HOXC13 for silencing its expression (siHOXC13-1 to siHOXC13-5), including negative control (siNC), were synthesized by GENERAL BIOL (Anhui, China). Cells (1 × 10^6^ cells/mL) were seeded in 6-well culture plate and transiently transfected with 2 mL OPTI-MEM medium (Gibco, USA) using siRNA transfection reagent (Santa Cruz, USA). siHOXC13-1 sequences: 5′-GG UGACGACCUGUCCUCUATT-3′ (forward) and 5′-UAGAGGACAGGUCGUCAC CTT-3′ (reverse). siHOXC13-2 sequences: 5′-CGUCGAAGGCUACCAGCACTT-3′ (forward) and 5′-GUGCUGGUAGCCUUCGACGTT-3′ (reverse). siHOXC13-3 sequences: 5′-AGGAAUACGCGGCUAGCAATT-3′ (forward) and 5′-UUGCUAGC CGCGUAUUCCUTT-3′ (reverse). siHOXC13-4 sequences: 5′-GCCUUAUGUACG UCUAUGATT-3′ (forward) and 5′-UCAUAGACGUACAUAAGGC-3′ (reverse). siHOXC13-5 sequences: 5′-GAAGAAGGUGGUCAGCAAATT-3′ (forward) and 5′-U UUGCUGACCACCUUCUUCTT-3′ (reverse). siNC sequences: 5′-UUCUCCGAAC GUGUCACGUTT-3′ (forward) and 5′-ACGUGACACGUUCGGAGAATT-3′ (reverse).

### Cell proliferation assay

After transfection for 12 h, cells (1 × 10^3^ cells/well) were seeded into 96-well culture plate with 100 μL serum medium. After incubation for 6, 12, 24 and 48 h, the OD values of cells at 450 nm were detected with 10 μL CCK-8 reagent (Beyotime, China). Calculated and plotted the cell proliferation curve based on CCK-8 method.

### Cell apoptosis assay

After transfection for 48 h, cells were collected and the cell suspension was prepared with 1 × binding buffer and adjusted to 1 × 10^6^ cells/tube, then 5 μL of Annexin V-APC and 10 μL of 7-AAD reagent were added, vortexed and mixed, and incubated for 15 min at room temperature (24 °C) and protected from light. Finally, 485 μL of pre-chilled 1 × binding buffer was added to each tube and resuspended, and the data were analyzed by flow cytometry with NovoExpress software.

### Cell migration assay

After transfection for 12 h, cells (5 × 10^5^ cells/well) were seeded into 6-well culture plate with 2 mL serum medium. Parallel lines were drawn at the bottom of the culture plate with the pipette tip, and the fallen cells were rinsed with PBS. The migrating cells at 0 and 48 h were imaged and the migration distances were calculated by Image J v1.8.0 software.

### Cell invasion assay

A double cavity transmission system with an 8 μm pore was used in this assay (the upper chamber was pre-added with 80 μL matrix solution and incubated at 37 ℃ for 1 h). After transfection for 12 h, cells (5 × 10^4^ cells/well) were seeded into the upper chamber of the inserts with 200 μL serum-free medium, and the lower chamber was filled with 500 μL serum medium. After invasion for 48 h, inserts were fixed with 100% methanol and then stained with 0.1% crystal violet (Biosharp, China). The invading cells on the inserts were imaged and counted by Image J v1.8.0.

### Cell viability assay

Cells (1 × 10^3^ cells/well) were seeded into 96-well culture plate with 100 μL serum medium for 24 h. Then 1, 5, 10, 20, 40, 60, 80 and 100 μM specific cGAS inhibitor RU.521 (MCE, USA) were added and cultured for 48 h, respectively. The OD values of cells at 450 nm were detected with 10 μL CCK-8 reagent.

### Immunofluorescence (IF) staining

Cells were fixed with 4% PFA for 15 min and permeabilized with 0.5% Triton X-100 (Sigma, USA) for 30 min. Then, dsDNA antibody (1:1000, Abcam, USA) was added to incubate the cells at 4 °C overnight. After washing three times, samples were incubated with secondary antibody-Alexa fluor 488 (1:200, Abcam, USA) for 1 h at room temperature and counterstained with DAPI (Sigma, USA). The staining was observed and imaged under fluorescence microscope.

### Quantitative reverse transcriptase PCR (qRT-PCR) assay

Total RNA was extracted from cells and tissues using Trizol reagent (BioTeke, China). After determining the concentration and purity of RNA samples, RNA reverse transcription was performed according to the steps of reverse transcription kit (Vazyme, China). The synthetic cDNAs were used as template for fluorescence detection using SYBR Green qPCR detection kit (Biosharp, China). The reaction procedure was as follows: pre-denaturation at 95 °C for 5 min, followed by 40 cycles of denaturation at 95 °C for 30 s, annealing at 60 °C for 30 s, and extension at 72 °C for 15 s. The results were quantified using the relative quantitative 2^−∆∆CT^ method. HOXC13 primer sequences: 5′-AAGGAATACGCGGCTAGCAA-3′ (forward) and 5′-GGAGATGAGGCGCTTTCGAT-3′ (reverse). IFN-β primer sequences: 5′-GACG CCGCATTGACCATCT-3′ (forward) and 5′-CCTTGGCCTTCAGGTAATGCAG-3′ (reverse). CCL2 primer sequences: 5′-CTCGCTCAGCCAGATGCAAT-3′ (forward) and 5′-TTGGGTTTGCTTGTCCAGGT-3′ (reverse). CCL5 primer sequences: 5′-CCT GTATGACTCCCGGCTGAA-3′ (forward) and 5′-AAGCCTCCCAAGCTAGGACA A-3′ (reverse). CXCL10 primer sequences: 5′-TGCCATTCTGATTTGCTGCCTT-3′ (forward) and 5′-GGACAAAATTGGCTTGCAGGAAT-3′ (reverse). ACTB primer sequences: 5′-TGGCACCAGCACAATGAA-3′ (forward) and 5′-CTAAGTCATAGTC CGCCTAGAAGCA-3′ (reverse).

### Western blot (WB) assay

Nucleoplasmic separation of cells according to the kit instruction (Beyotime, China). Cells were lysed in RIPA lysis buffer (Sigma, USA) supplemented with 0.1% protease-inhibitor (Sigma, USA). The isolated proteins (40 µg) were separated on 12% SDS gels and electrotransferred to polyvinylidene difluoride membranes. Then, incubation for 16 h with primary antibodies against AR (1:1500, CST, USA), HOXC13 (1:1000, ABclonal, China), γH2AX (1:1000, ABclonal, China), cGAS (1:1000, ABclonal, China), p(Ser366)-STING (1:1000, CST, USA), STING (1:1000, CST, USA), p(Ser386)-IRF3 (1:1000, CST, USA), IRF3 (1:1000, CST, USA), H3 (1:1000, Beyotime, China) and ACTB (1:1000, BOSTER, China). The membrane was washed and incubated in horseradish peroxidase-conjugated secondary antibody. Antibody-bond protein bands were assayed using a chemiluminescent luminol enhancer solution (YEASEN, China). The gel imaging system was used for imaging analysis.

### Statistical analysis

All experiments were performed in triplicate. Statistical analysis was performed by SPSS v18.0 software. Experimental data were presented as mean ± standard deviation (SD). Comparison between multiple groups was made using one-way ANOVA (Tukey's posthoc test). P < 0.05 was considered statistically significant.

## Results

### Gene cluster (HOXC10, HOXC12 and HOXC13) with unique clinical features in PCa

**Fig. 1:**
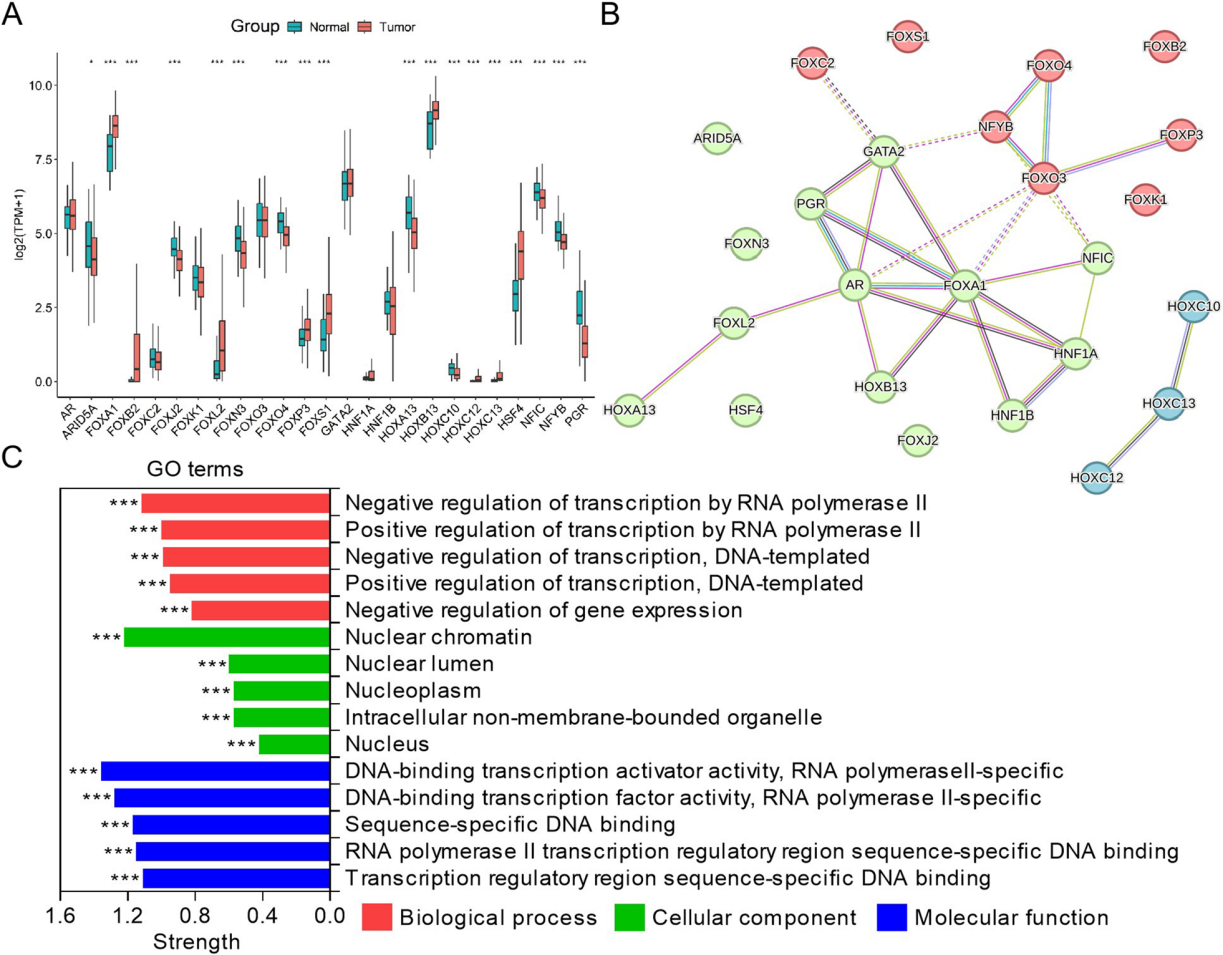
Expression properties of 25 TFs in PCa. **A** Expression of 25 TFs in PCa tissues (n = 498) and paracancerous tissues (n = 52). **B** Interaction and clustering among 25 TFs. **C** GO enrichment of 25 TFs. (*P < 0.05, **P < 0.01, ***P < 0.001)

**Fig. 2:**
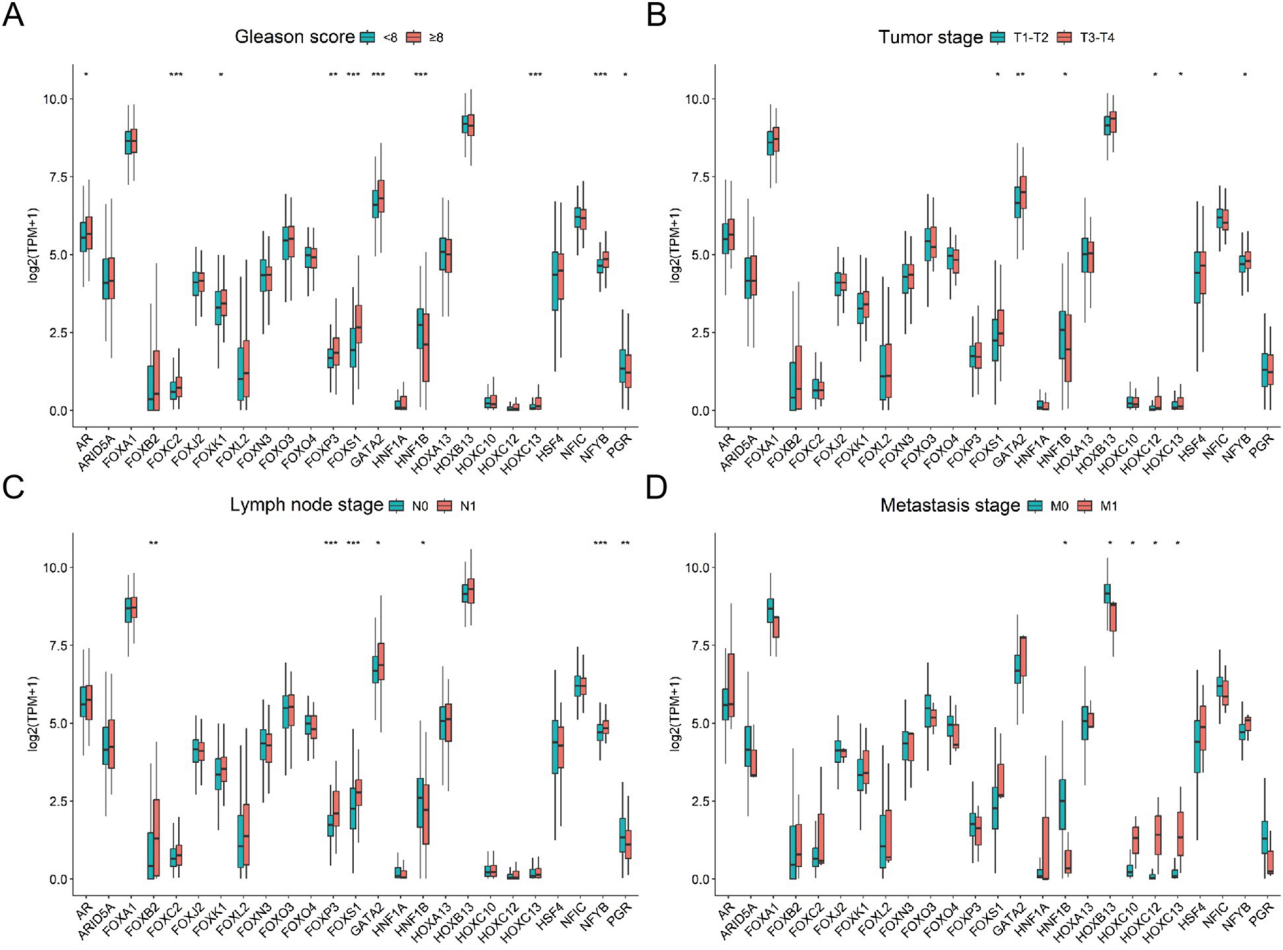
Correlation between 25 TFs and clinical features in PCa. **A** Expression of 25 TFs in PCa patients with GS < 8 (n = 285) and GS ≥ 8 (n = 196). **B** Expression of 25 TFs in PCa patients with T1–T2 stage (n = 186) and T3–T4 stage (n = 288). **C** Expression of 25 TFs in PCa patients with N0 stage (n = 337) and N1 stage (n = 75). **D** Expression of 25 TFs in PCa patients with M0 stage (n = 440) and M1 stage (n = 3). (*P < 0.05, **P < 0.01, ***P < 0.001)

To understand the expression properties of 25 TFs in PCa and their correlation with clinical features, we used bioinformatics to comprehensively analyze their role in PCa progression. We found that 25 TFs were aberrantly expressed in PCa, with FOXA1, FOXB2, FOXL2, FOXP3, FOXS1, HOXB13, HOXC12, HOXC13 and HSF4 being highly expressed in tumor tissues, while ARID5A, FOXJ2, FOXN3, FOXO4, HOXA13, HOXC10, NFIC, NFYB and PGR were lowly expressed (Fig. 1A). PPI analysis of 25 TFs revealed that they could be classified into three clusters, with close interaction between green cluster (AR, ARID5A, FOXA1, FOXJ2, FOXL2, FOXN3, GATA2, HNF1A, HNF1B, HOXA13, HOXB13, HSF4, NFIC and PGR) and red cluster (FOXB2, FOXC2, FOXK1, FOXO3, FOXO4, FOXP3, FOXS1 and NFYB), while blue cluster (HOXC10, HOXC12 and HOXC13) was independent, suggesting that blue cluster may have a unique regulatory pattern in PCa progression (Fig. 1B). Functional enrichment revealed that 25 TFs were mainly enriched in nuclear chromatin and involved in positive or negative transcriptional regulation of genes (Fig. 1C). Further analysis revealed that the expression of 25 TFs was almost positively correlated, with FOXA1 being the most strongly correlated with HOXB13, in addition to HOXC12 being highly positively correlated with HOXC13 in the blue cluster (Additional file 1: Fig. S1). Clinical features demonstrated that 25 TFs were aberrantly expressed in different pathological states, among which HOXC12 and HOXC13 were highly expressed in advanced T stage and M stage, but not with N stage (Fig. 2B–D). HOXC13 was also highly expressed in high GS (Fig. 2A). These results suggest a potential regulatory role for gene cluster (HOXC10, HOXC12 and HOXC13) in PCa progression.

### High HOXC13 expression has a worse prognosis for PCa patients

**Fig. 3:**
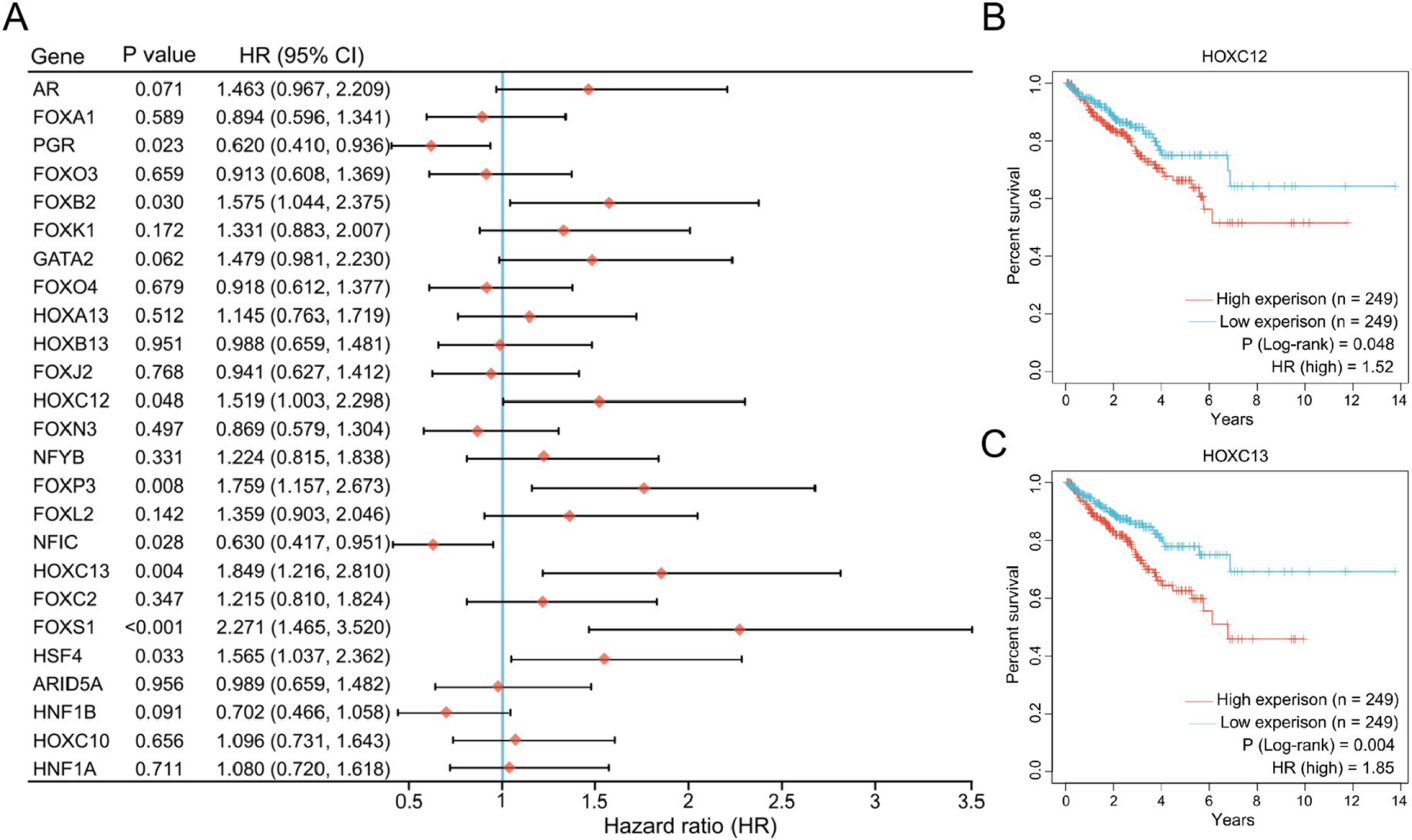
Prognostic characterization of 25 TFs in PCa. **A** Risk levels of 25 TFs in PCa. **B** OS of PCa patients with low (n = 249) and high (n = 249) HOXC13 expression (P = 0.048, HR = 1.52). **C** OS of PCa patients with low (n = 249) and high (n = 249) HOXC13 expression (P = 0.004, HR = 1.85)

**Fig. 4:**
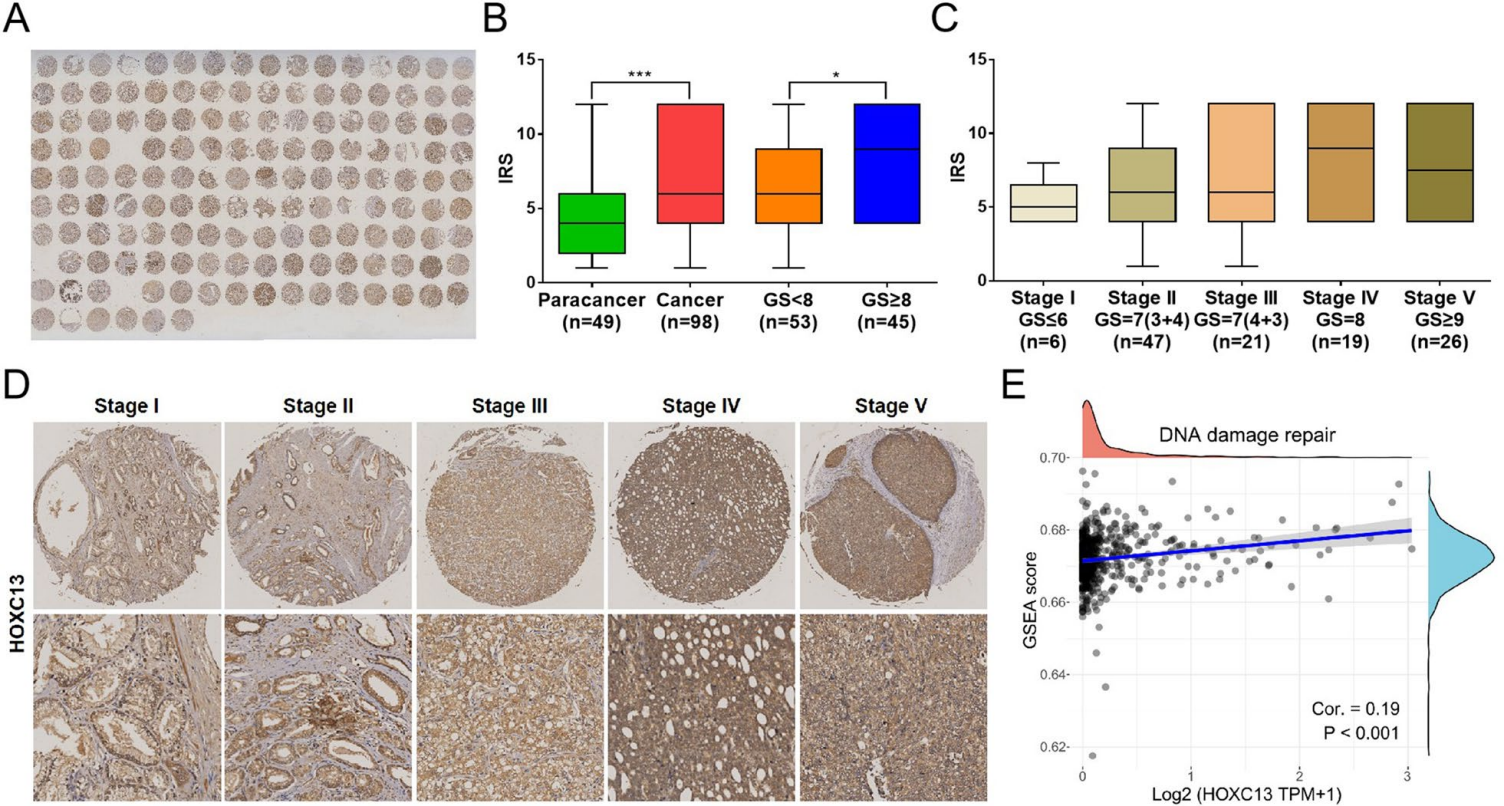
Expression of HOXC13 in PCa tissues. **A** Full view of IHC staining for HOXC13 expression (147 cases available after excluding 3 cases without staining). **B** Expression statistics of HOXC13 in paracancerous tissues (n = 49) and PCa tissues (n = 98) and PCa tissues with GS < 8 (n = 53) and GS ≥ 8 (n = 45). **C** Expression statistics of HOXC13 in PCa tissue with stage I (n = 6), stage II (n = 47), stage III (n = 21), stage IV (n = 19) and stage V (n = 26). **D** Expression of HOXC13 in PCa tissues with different tumor stages (stage I to stage V). **E** Correlation between HOXC13 expression and DNA damage repair levels (r = 0.19, P < 0.001). (*P < 0.05, ***P < 0.001)

Further, we analyzed the effects of 25 TFs on OS of PCa patients. Prognostic analysis showed that FOXB2, HOXC12, FOXP3, HOXC13, FOXS1 and HSF4 were risk factors for PCa, while PGR and NFIC were protective factors (Fig. 3A). Although both HOXC12 and HOXC13 were risk factors for PCa, patients with high HOXC13 expression have a worse prognosis (Fig. 3B and C). Next, we analyzed the expression properties of HOXC13 in PCa using tissue microarray. IHC staining showed that HOXC13 was highly expressed in tumor tissues compared to paracancerous tissues and was also highly expressed in tumor tissues with high GS (Fig. 4A and B). Further classifying PCa patients into five categories (stage I to stage V) based on pathological grade, we found that HOXC13 was highly expressed in patients with high stage, and interestingly, HOXC13 was most highly expressed in patients with stage IV (Fig. 4C and D). Since the HOX family is widely involved in cellular DNA repair pathways, we analyzed the correlation between HOXC13 expression and DNA damage repair levels in PCa. Correlation analysis demonstrated that HOXC13 expression was positively correlated with DNA damage repair levels, suggesting that high HOXC13 expression promotes DNA damage repair in PCa cells (Fig. 4E).

### Silencing HOXC13 exerts anti-tumor effects in vitro

**Fig. 5:**
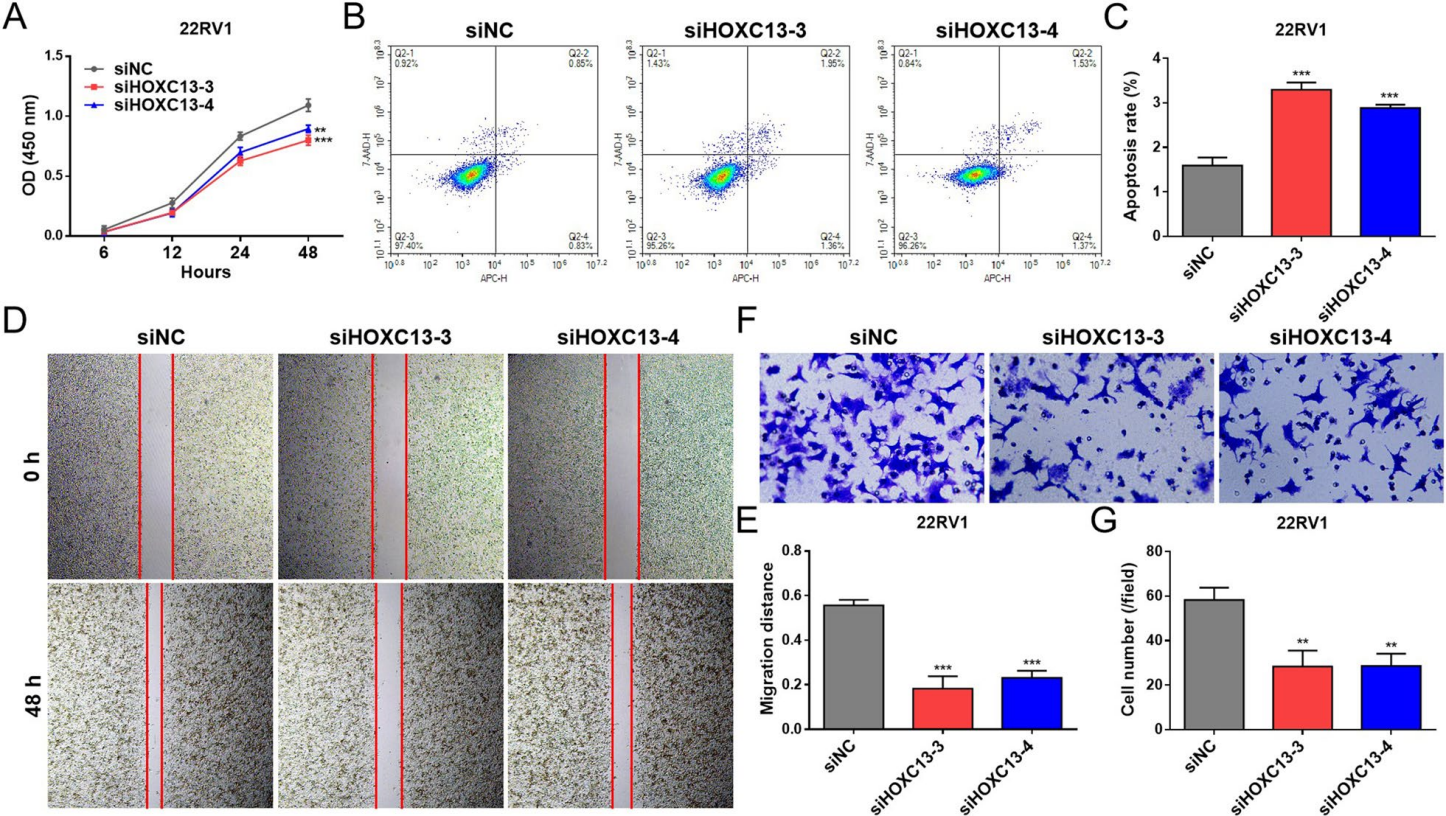
Effects of silencing HOXC13 on 22RV1 cell function. **A** Cell proliferation levels at 6, 12, 24 and 48 h. **B** Cell apoptosis levels at 48 h. **C** Statistics of cell apoptosis levels. **D** Cell migration levels at 48 h. **E** Statistics of cell migration levels. **F** Cell invasion levels at 48 h. **G** Statistics of cell invasion levels. (*P < 0.05, **P < 0.01, ***P < 0.001)

To further clarify the role of HOXC13 in PCa, we performed reverse validation by gene silencing. Since HOXC13 is a key driver of CRPC-AR subtype, we proposed to select PCa cell lines with high or null AR expression for the study to observe whether the effect of HOXC13 on PCa cell function possesses AR expression dependence. The transcriptional expression levels of HOXC13 in different PCa cell lines were observed using THE HUMAN PROTEIN ATLAS (https://www.proteinatlas.org/), and combined with protein expression validation (Additional file 2: Fig. S2A and B), the 22RV1 (HOXC13^+^/AR^+^) and DU145 (HOXC13^+^/AR^−^) cell lines were finally selected for subsequent experiments. To eliminate false positive results caused by off-target effects, we selected two siRNAs with the best interference effect for comparison. Combining the results of qRT-PCR and WB, we found that both siHOXC13-3 and HOXC13-4 had a more stable interference effect in 22RV1 and DU145 cells, which can be used for subsequent experiments (Additional file 3: Fig. S3A–F). Cell function assays showed that silencing HOXC13 significantly inhibited 22RV1 cell proliferation, migration and invasion, and promoted cell apoptosis (Fig. 5A–G). The same results were shown for DU145 cell function (Additional file 4: Fig. S4A–G). These results indicate that the effects of HOXC13 on PCa cell function are not dependent on AR expression. HOXC13 plays an important mediating role in PCa development, suggesting that HOXC13 is an oncogene for PCa.

### Silencing HOXC13 induces cellular DNA damage and activates cGAS/STING/ IRF3 pathway

**Fig. 6:**
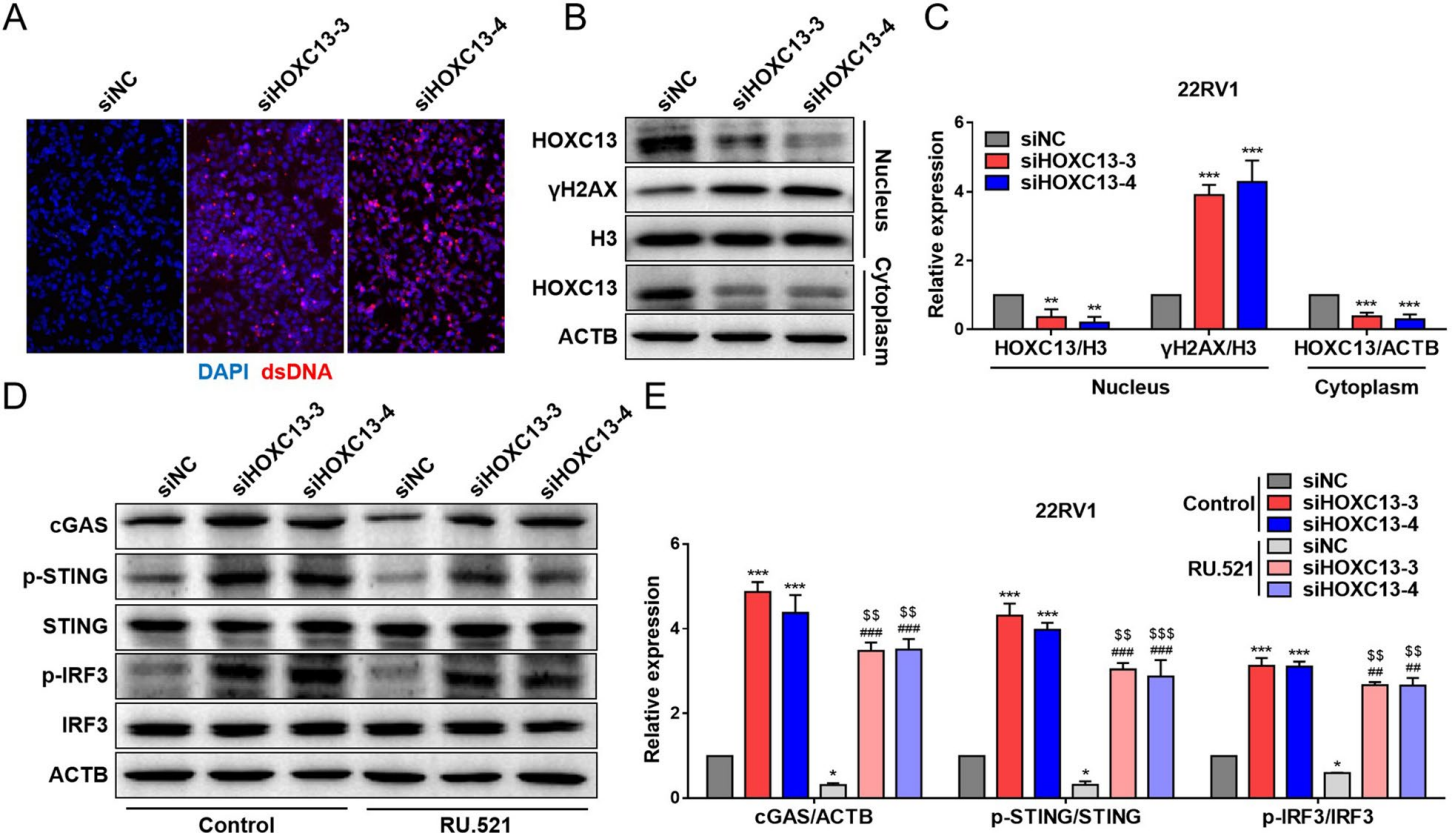
Effects of silencing HOXC13 on DNA damage-induced cGAS/STING/IRF3 pathway in 22RV1 cells. **A** Accumulation of dsDNA in cells. **B** Protein bands for nucleus and cytoplasm. **C** Expression statistics of HOXC13 and γH2AX proteins. **D** Protein bands for cytoplasm. **E** Expression statistics of cGAS, p-STING, STING, p-IRF3 and IRF3 proteins. (vs. siNC group: *P < 0.05, **P < 0.01, ***P < 0.001; vs. siHOXC13-3 group: ^###^P < 0.001; vs. siHOXC13-4 group: ^$$^P < 0.01, ^$$$^P < 0.001)

Since HOXC13 mediates cellular DNA damage repair, we speculate that silencing HOXC13 may disrupt DNA repair mechanisms and exacerbate DNA damage. When DNA damage reaches a certain level, numerous broken double- stranded DNA (dsDNA) is released into cytoplasm and accumulates. cGAS/STING/IRF3, as a novel DNA damage response pathway, can be activated by cytoplasmic dsDNA to regulate cell function and immune activity. IF staining revealed that silencing HOXC13 significantly promoted dsDNA accumulation in 22RV1 cells (Fig. 6A). When cellular DNA is damaged, serine 139 on H2AX undergoes phosphorylation modification to form γH2AX, which is a biomarker to measure the degree of DNA damage. WB results further demonstrated that silencing HOXC13 significantly inhibited its nuclear translocation and up-regulated γH2AX in 22RV1 cells (Fig. 6B and C). To validate the mediated relationship between HOXC13 and cGAS/STING/IRF3 pathway, reverse proof was performed using the specific cGAS inhibitor RU.521. Pre-experiments showed that 20 μM RU.521 was not toxic to 22RV1 and DU145 cells and could effectively inhibit cGAS expression (Additional file 5: Fig. S5A–D). Therefore, this dose was selected for subsequent experiments. Pathway validation demonstrated that RU.521 (a specific cGAS inhibitor) could attenuate silencing HOXC13-induced cGAS up-regulation and STING and IRF3 phosphorylation in 22RV1 cells (Fig. 6D and E). Similarly, we obtained consistent results on DU145 cell experiments (Additional file 6: Fig. S6A–E). These results indicated that silencing HOXC13 induces DNA damage and activates cGAS/STING/IRF3 pathway in PCa cells.

### High HOXC13 expression suppresses anti-tumor immune response

**Fig. 7:**
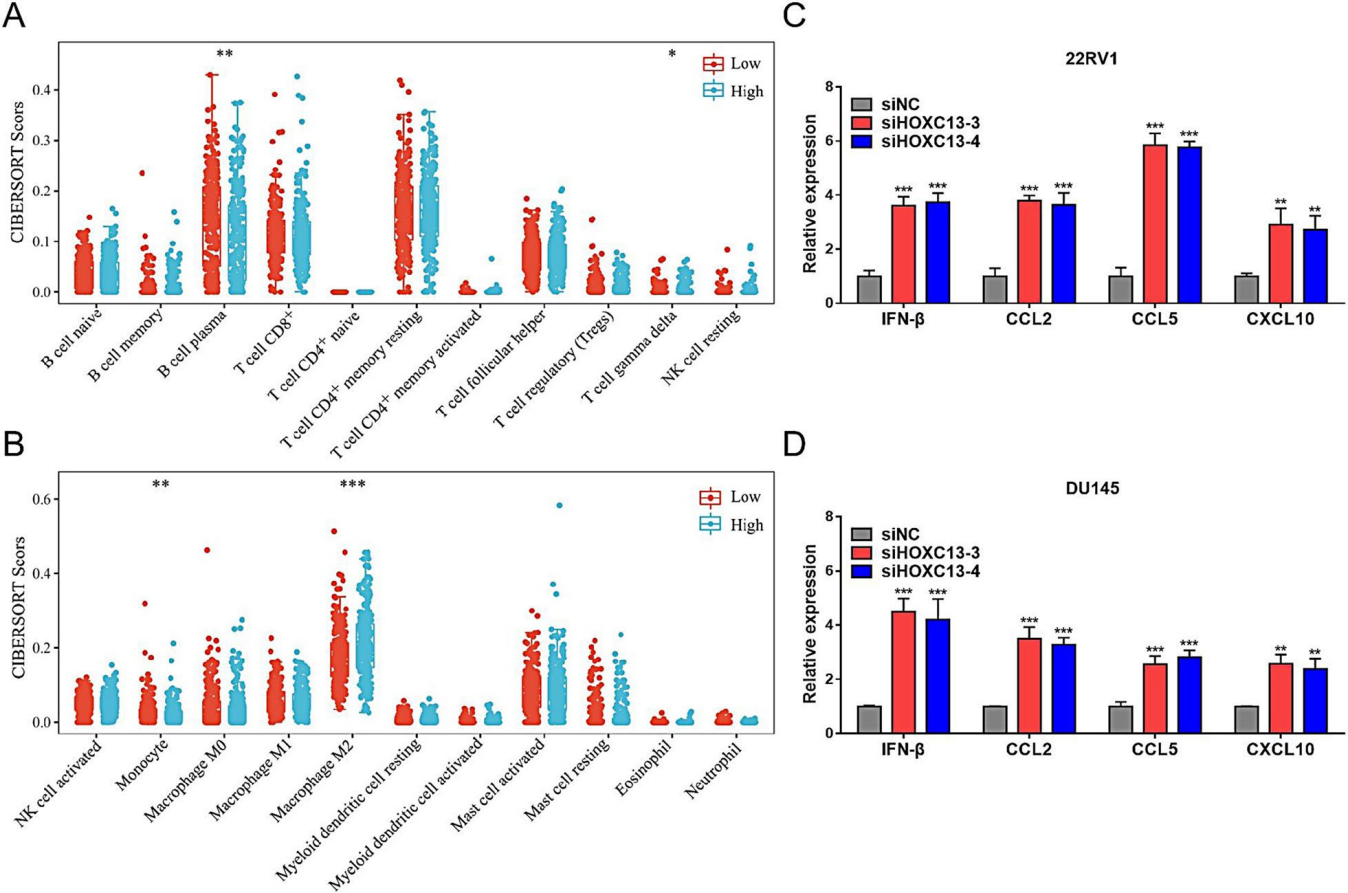
Correlation between HOXC13 expression and immune infiltration in PCa. **A**, **B** Expression of 22 immune cells in low (n = 249) and high (n = 249) HOXC13 expression groups. **C** Expression statistics of IFN-β, CCL2, CCL5 and CXCL10 transcripts in 22RV1 cells. **D** Expression statistics of IFN-β, CCL2, CCL5 and CXCL10 transcripts in DU145 cells. (*P < 0.05, **P < 0.01, ***P < 0.001)

cGAS/STING/IRF3 pathway is known to play a critical role in innate immune response and anti-tumor immunity. Therefore, whether HOXC13 mediates the alteration of TIME in PCa aroused our curiosity. Immune infiltration analysis revealed that various immune cells were infiltrated in TIME and that PCa patients with high HOXC13 expression were accompanied by low infiltration of tumor-killing immune cells (γδ T cells and plasma cells) and monocytes, while promoting high infiltration of tumor promoting immune cells (M2 macrophages) (Fig. 7A and B). To verify the effects of HOXC13 on immune response, we examined the transcriptional expression levels of IFN-β, a downstream gene of IRF3, and some immune-related chemokines (CCL2, CCL5 and CXCL10). qRT-PCR results showed that silencing HOXC13 significantly up-regulated IFN-β, CCL2, CCL5 and CXCL10 in 22RV1 and DU145 cells (Fig. 7C and D). These results suggest that HOXC13 can remodel the TIME in PCa, that high HOXC13 expression suppresses anti-tumor immune response, and that HOXC13 may be a potential target for immunotherapy.

## Discussion

PCa development involves dysregulation of key TFs expression or disruption of transcriptional network following gene mutation [22]. Based on 25 key TFs in CRPC-AR, we comprehensively assessed their characterization in PCa progression. Bioinformatics results revealed that 25 TFs were aberrantly expressed in PCa, particularly in FOX family (FOXA1, FOXO3, FOXB2, FOXK1, FOXO4, FOXJ2, FOXN3, FOXP3, FOXL2, FOXC2 and FOXS1) and HOX family (HOXA13, HOXB13, HOXC10, HOXC12 and HOXC13). Involvement of FOX family in PCa formation and metastasis has been reported in numerous cases, of which FOXA1 is essential in regulating AR-mediated tumor development, and other FOX members have been confirmed to play regulatory roles in progression of various cancers [23]. PPI analysis also indicated the central role of FOX family in 25 TFs. However, we were surprised to find HOXC10, HOXC12 and HOXC13 in HOX family as an independent cluster whose role in PCa has rarely been reported, which aroused our strong concern.

HOX gene encodes a family of transcription factors containing homologous structural domains (containing four clusters A, B, C and D) that play important roles in early embryo, including establishment of cellular and tissue identity, as well as regulation of cellular proliferation, differentiation and survival [24]. HOX proteins have non-transcriptional activity and are involved in regulation of various processes, such as DNA replication and repair, mRNA translation and protein degradation [25]. Numerous studies have confirmed that most of HOX genes are oncogenic and support malignant phenotypes, such as cervical cancer, colon cancer and esophageal cancer [26–28]. Further analysis revealed that HOX family was closely associated with clinical features of PCa, among which HOXC13 was particularly prominent and highly correlated with GS, T stage and M stage. Tissue microarray demonstrated that HOXC13 was highly expressed in PCa tissues and was more overexpressed in patients with high pathological grade. Survival analysis also revealed that high HOXC13 expression was accompanied by worse prognosis. These results suggest that HOXC13 is an oncogene involved in regulation of PCa progression. Land et al. [29] demonstrated that HOXC13 may be able to differentiate between recurrent and non-recurrent PCa, but its mechanism of action is unknown. In addition, the association of HOXC13 with other cancers has been confirmed. In breast cancer, HOXC13 is specifically overexpressed and has a poor prognosis for patients and was strongly correlated with N stage and M stage [30]. In glioblastoma, HOXC13 is an important diagnostic and prognostic biomarker [31]. In vitro experiments further demonstrated the important regulatory role of HOXC13 in PCa cell function, silencing HOXC13 significantly inhibited cell proliferation, migration and invasion, and promoted cell apoptosis. These results are consistent with its role in other cancer cells. In non-small cell lung cancer, silencing HOXC13 counteracts HOXC-AS2 overexpression-induced increases in cell proliferation and migration and decreases in cell apoptosis [32]. In cervical cancer, HOXC13 promotes cell proliferation, migration, invasion and glycolysis by regulating β-catenin/c-Myc pathway [33]. These results suggest a high involvement of HOXC13 in PCa progression.

HOX family is important molecules that mediate DNA damage repair [34]. As a member of the HOX family, HOXC13 is most likely to be involved in DNA repair mechanism, as verified by gene-pathway correlation analysis. Further assays revealed that silencing HOXC13 promoted dsDNA accumulation and recruited γH2AX into nucleus. These results suggest that HOXC13 mediates DNA damage repair in PCa cells. Recent studies have revealed that the innate immune system pathway, cGAS/STING/IRF3, plays a critical role in DNA damage response and immune regulation in various cancers [35–37]. Silencing HOXC13 disrupts cellular DNA repair mechanisms, exacerbates DNA damage and releases dsDNA into cytoplasm. Numerous cytoplasmic dsDNA can be sensed by cGAS to recruit STING anchored in endoplasmic reticulum and triggers phosphorylation activity of IRF3, leading to downstream biological responses. Detection of cGAS/STING/IRF3 pathway-related molecules confirmed the above speculations. Our study establishes a link between HOXC13 and DNA damage-induced cGAS/STING/IRF3 pathway for the first time, which provides novel molecular targets for PCa therapy.

Clinically, ADT remains the basic treatment for advanced PCa. ADT drugs include androgen synthesis inhibitors (like Abiraterone), AR inhibitors (like Enzalutamide) and novel AR-targeted drugs (like Apalutamide) [38]. Although ADT is initially effective, it will inevitably progress to CRPC. For AR-dependent CRPC, ADT induces mutations in PCa cells so that they can continue to grow at extremely low concentrations of androgens. These variants include acquired high AR expression or mutation, androgen-independent AR activation, up-regulation of steroid synthase expression and sustained high AR expression in a non-glandular-dependent manner [39]. For AR-independent CRPC, which is inherently insensitive to ADT drugs, resistance can be maintained without AR expression relying on the activation of other signaling pathways (like PI3K/Akt/mTOR and Wnt/β-catenin pathways) [40, 41]. Therefore, ADT efficacy is limited by AR status. Our study found that silencing HOXC13 exerted anti-PCa effects in an AR-independent manner, suggesting a degree of superiority over ADT. Moreover, AR expression unaffected HOXC13-mediated cGAS/STING/IRF3 pathway activation. As the most active molecule in cGAS signaling, STING engages in the regulation of drug resistance in various cancers. For example, pharmacological STING activation is a potential alternative to overcome drug resistance in melanoma [42]. Recent studies have found that CRPC patients resistant to Abiraterone and Enzalutamide have higher IL-6 levels [43]. In addition, IL-6-induced STAT3 activation promotes CRPC transformation, ADT resistance and Gankyrin expression, leading to formation of the Gankyrin/NONO/AR/HMGB1/IL-6/STAT3 positive feedback signaling pathway, in which STAT3 is the most important transduction molecule [44, 45]. Interestingly, STING activation overcomes STAT3-mediated immunosuppression and adaptive resistance to PARP inhibition [46]. Although there is lack of evidence related to HOXC13-mediated ADT resistance, these studies suggest that it is feasible to inhibit ADT resistance based on activation of STING signaling. Overall, these studies provide guidance for clinical combination therapy (especially with ADT) targeting HOXC13 and its downstream molecules.

In recent years, immunotherapy by modulating the immune function of TME has shown favorable efficacy in various cancers, but limited efficacy in PCa [47]. Therefore, it is necessary to investigate the role of HOXC13 in regulating TIME in PCa. Immune infiltration analysis revealed that TIME was suppressed in PCa patients with high HOXC13 expression, accompanied by low infiltration of γδ T cells, plasma cells and monocytes, and high infiltration of M2 macrophages. γδ T cells are T cells that perform innate immune function, both killing cancer cells and recognizing cancer antigens. The immune response of γδ T cells is biased towards type I immunity and kills tumor cells mainly by producing granzyme B and perforin [48]. Further studies found that silencing HOXC13 significantly increased IFN-β, CCL2, CCL5 and CXCL10 production, which are downstream genes known to be transcriptionally activated by IRF3 and play important regulatory roles in the modulation of immune cell function [49–51]. γδ T cells can be activated by IFN-β and CXCL10 induced in TME to exert cytotoxic effects [52]. In addition, IFN-β can partially activate B cells to mediate plasma cell formation and participate in humoral immunity [53]. CCL2 and CCL5 can recruit monocytes to migrate toward tumor foci [54, 55]. In addition to phagocytosis, monocytes can be further induced to differentiate into macrophages, of which M2 macrophages are critical in assisting cancer cells to generate immune escape [56]. These results suggest that HOXC13 has promising application value in mediating anti-tumor immune response and can be used as a novel immunotherapeutic target.

In conclusion, our study revealed HOXC13 as a novel pro-PCa gene that regulates PCa progression by mediating DNA damage-induced cGAS/STING/IRF3 pathway. In addition, HOXC13 can remodel TIME by regulating transcription of immune factors IFN-β, CCL2, CCL5 and CXCL10.

### Supplementary Information

**Additional file 1: Figure S1.** Co-expression correlation among 25 TFs.

**Additional file 2: Figure S2.** Expression of AR and HOXC13 in 22RV1 and DU145 cells. (A) Protein bands for cells. (B) Expression statistics of AR and HOXC13 proteins. (***P < 0.001).

**Additional file 3: Figure S3.** Validation of silencing efficiency for siRNAs targeting HOXC13. (A) Expression statistics of HOXC13 transcript in 22RV1 cells. (B) Protein bands for 22RV1 cells. (C) Expression statistics of HOXC13 protein in 22RV1 cells. (D) Expression statistics of HOXC13 transcript in DU145 cells. (E) Protein bands for DU145 cells. (F) Expression statistics of HOXC13 protein in DU145 cells. (*P < 0.05, **P < 0.01, ***P < 0.001).

**Additional file 4: Figure S4.** Effects of silencing HOXC13 on DU145 cell function. (A) Cell proliferation levels at 6, 12, 24 and 48 h. (B) Cell apoptosis levels at 48 h. (C) Statistics of cell apoptosis levels. (D) Cell migration levels at 48 h. (E) Statistics of cell migration levels. (F) Cell invasion levels at 48 h. (G) Statistics of cell invasion levels. (*P < 0.05, **P < 0.01, ***P < 0.001).

**Additional file 5: Figure S5.** Screening for optimal dose of the specific cGAS inhibitor RU.521. (A) Effect of different doses of RU.521 on 22RV1 cell viability. (B) Effect of different doses of RU.521 on DU145 cell viability. (C) Protein bands for cells. (D) Expression statistics of cGAS proteins. (vs. 0 μM RU.521 group in 22RV1 cells: *P < 0.05, **P < 0.01, ***P < 0.001; vs. 0 μM RU.521 group in DU145 cells: ^#^P < 0.05, ^##^P < 0.01, ^###^P < 0.001).

**Additional file 6: Figure S6.** Effects of silencing HOXC13 on DNA damage-induced cGAS/STING/IRF3 pathway in DU145 cells. (A) Accumulation of dsDNA in cells. (B) Protein bands for nucleus and cytoplasm. (C) Expression statistics of HOXC13 and γH2AX proteins. (D) Protein bands for cytoplasm. (E) Expression statistics of cGAS, p-STING, STING, p-IRF3 and IRF3 proteins. (vs. siNC group: *P < 0.05, **P < 0.01, ***P < 0.001; vs. siHOXC13-3 group: ^###^P < 0.001; vs. siHOXC13-4 group: ^$$^P < 0.01, ^$$$^P < 0.001).

## Data Availability

The data presented in this study are available on request from the corresponding authors.

## Abbreviations

PCa: Prostate cancer
PRAD: Prostate adenocarcinoma
CRPC: Castration-resistant prostate cancer
ADT: Androgen deprivation therapy
TFs: Transcription factors
TME: Tumor microenvironment
TIME: Tumor immune microenvironment
ATAC-seq: Transposase-accessible chromatin sequencing
RNA-seq: RNA sequencing
TCGA: The Cancer Genome Atlas
cBioPortal: CBio Cancer Genomics Portal
FPKM: Fragments per kilobase per million
TPM: Transcripts per million
GS: Gleason score
T: Tumor
N: Lymph node
M: Metastasis
dsDNA: Broken double-stranded DNA
PPI: Protein–protein interaction
GO: Gene ontology
FDR: False discovery rate
OS: Overall survival
HR: Hazard ratio
CI: Confidence interval
siRNAs: Small interfering RNAs
IHC: Immunohistochemistry
IF: Immunofluorescence
qRT-PCR: Quantitative reverse transcriptase PCR
WB: Western blot
AR: Androgen receptor
HOXC13: Homeobox C13
γH2AX: Gamma H2A histone family member X
H3: Histone H3
cGAS: Cyclic GMP-AMP synthase
STING: Stimulator of interferon genes
p-STING: Phosphorylated STING
IRF3: Interferon regulatory factor 3
p-IRF3: Phosphorylated IRF3
IFN-β: Interferon-beta
CCL2: C-C motif chemokine ligand 2
CCL5: C-C motif chemokine ligand 5
CXCL10: C-X-C motif chemokine ligand 10
ACTB: Actin beta
