# Supplementary figures and images for: Silencing HOXC13 exerts anti-prostate cancer effects by inducing DNA damage and activating cGAS/STING/IRF3 pathway
Source: J Transl Med. 2023 Dec 6;21:884. doi: 10.1186/s12967-023-04743-x (PMC10701956; doi:10.1186/s12967-023-04743-x)

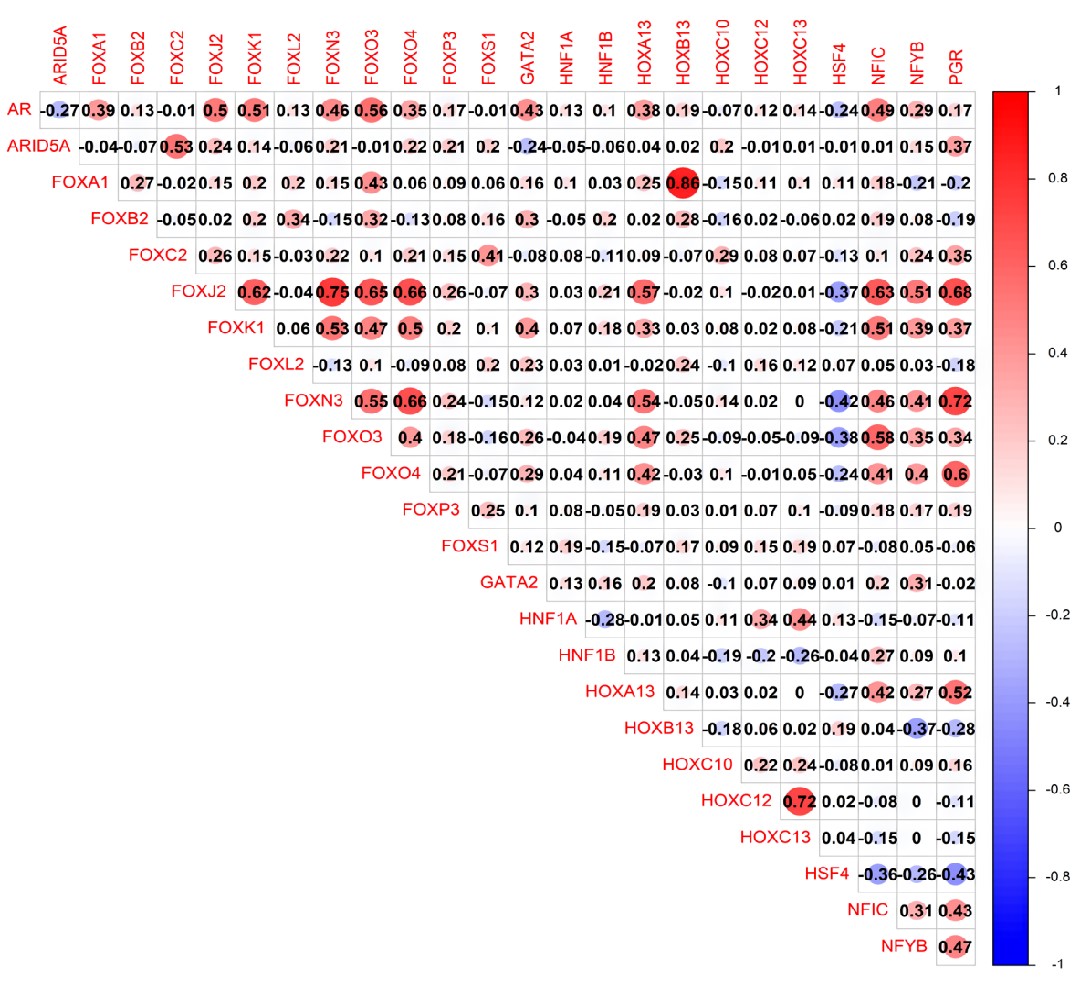

Supplement: Supplementary file 1 — Additional file 1: Figure S1. Co-expression correlation among 25 TFs. [file 12967_2023_4743_MOESM1_ESM.jpg]

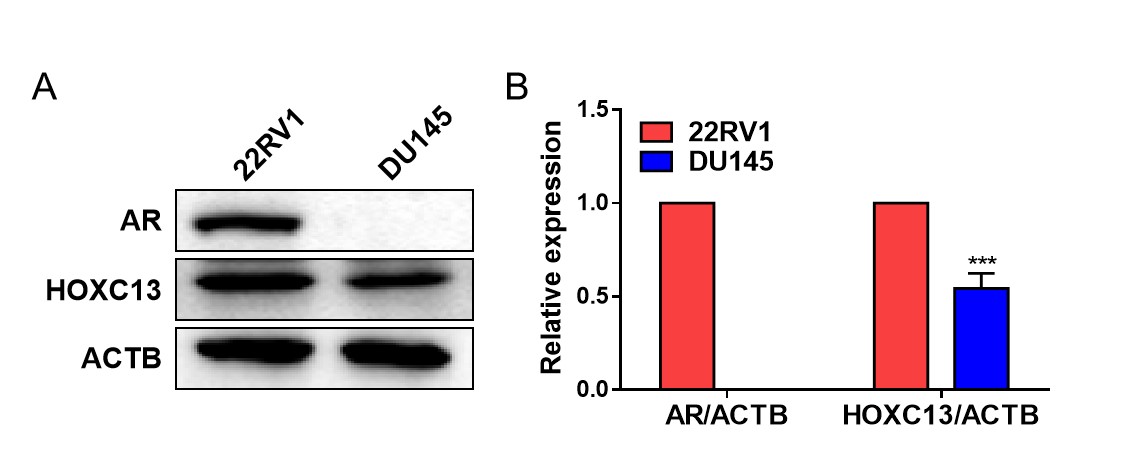

Supplement: Supplementary file 2 — Additional file 2: Figure S2. Expression of AR and HOXC13 in 22RV1 and DU145 cells. (A) Protein bands for cells. (B) Expression statistics of AR and HOXC13 proteins. (***P < 0.001). [file 12967_2023_4743_MOESM2_ESM.jpg]

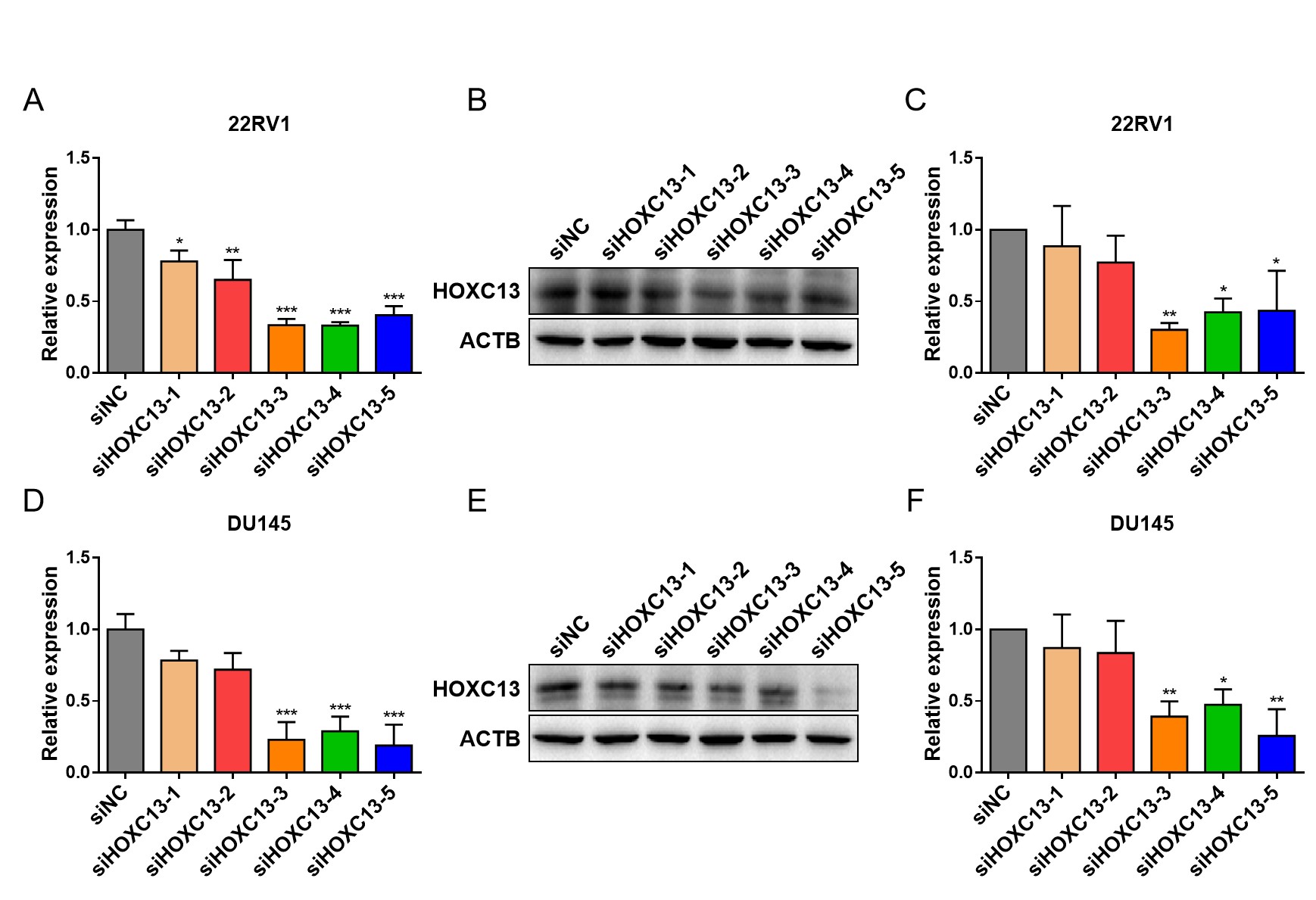

Supplement: Supplementary file 3 — Additional file 3: Figure S3. Validation of silencing efficiency for siRNAs targeting HOXC13. (A) Expression statistics of HOXC13 transcript in 22RV1 cells. (B) Protein bands for 22RV1 cells. (C) Expression statistics of HOXC13 protein in 22RV1 cells. (D) Expression statistics of HOXC13 transcript in DU145 cells. (E) Protein bands for DU145 cells. (F) Expression statistics of HOXC13 protein in DU145 cells. (*P < 0.05, **P < 0.01, ***P < 0.001). [file 12967_2023_4743_MOESM3_ESM.jpg]

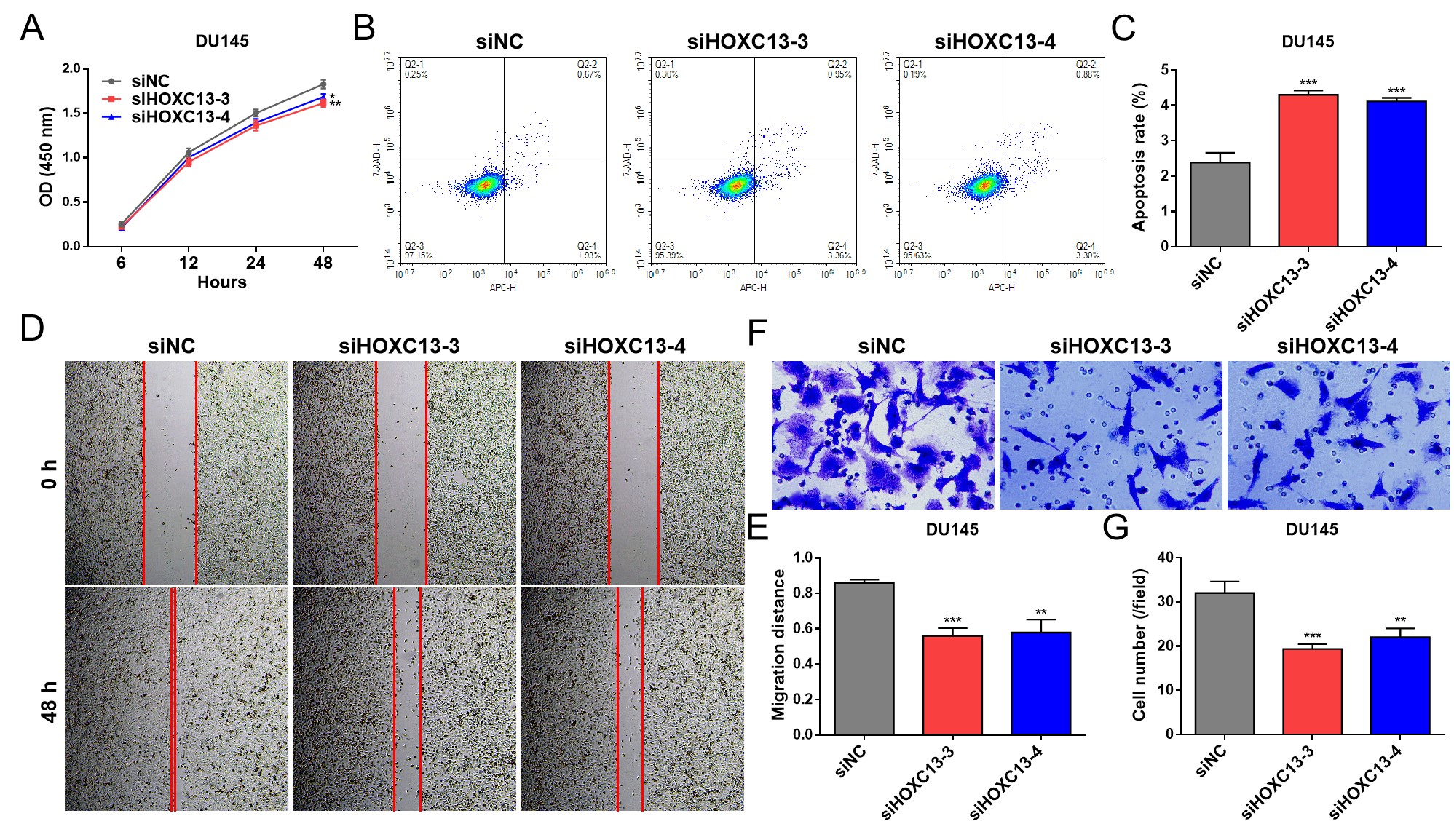

Supplement: Supplementary file 4 — Additional file 4: Figure S4. Effects of silencing HOXC13 on DU145 cell function. (A) Cell proliferation levels at 6, 12, 24 and 48 h. (B) Cell apoptosis levels at 48 h. (C) Statistics of cell apoptosis levels. (D) Cell migration levels at 48 h. (E) Statistics of cell migration levels. (F) Cell invasion levels at 48 h. (G) Statistics of cell invasion levels. (*P < 0.05, **P < 0.01, ***P < 0.001). [file 12967_2023_4743_MOESM4_ESM.jpg]

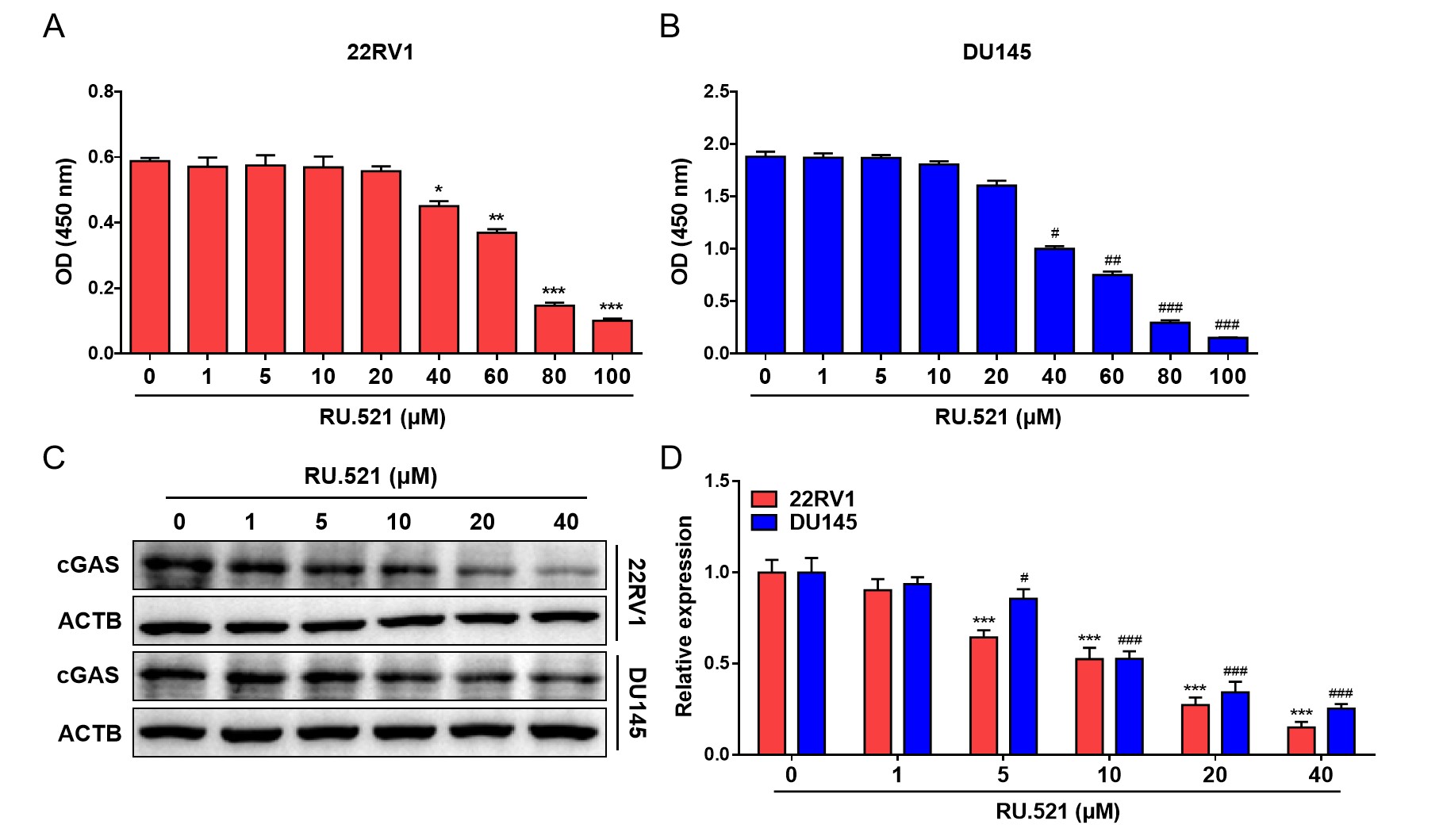

Supplement: Supplementary file 5 — Additional file 5: Figure S5. Screening for optimal dose of the specific cGAS inhibitor RU.521. (A) Effect of different doses of RU.521 on 22RV1 cell viability. (B) Effect of different doses of RU.521 on DU145 cell viability. (C) Protein bands for cells. (D) Expression statistics of cGAS proteins. (vs. 0 μM RU.521 group in 22RV1 cells: *P < 0.05, **P < 0.01, ***P < 0.001; vs. 0 μM RU.521 group in DU145 cells: #P < 0.05, ##P < 0.01, ###P < 0.001). [file 12967_2023_4743_MOESM5_ESM.jpg]

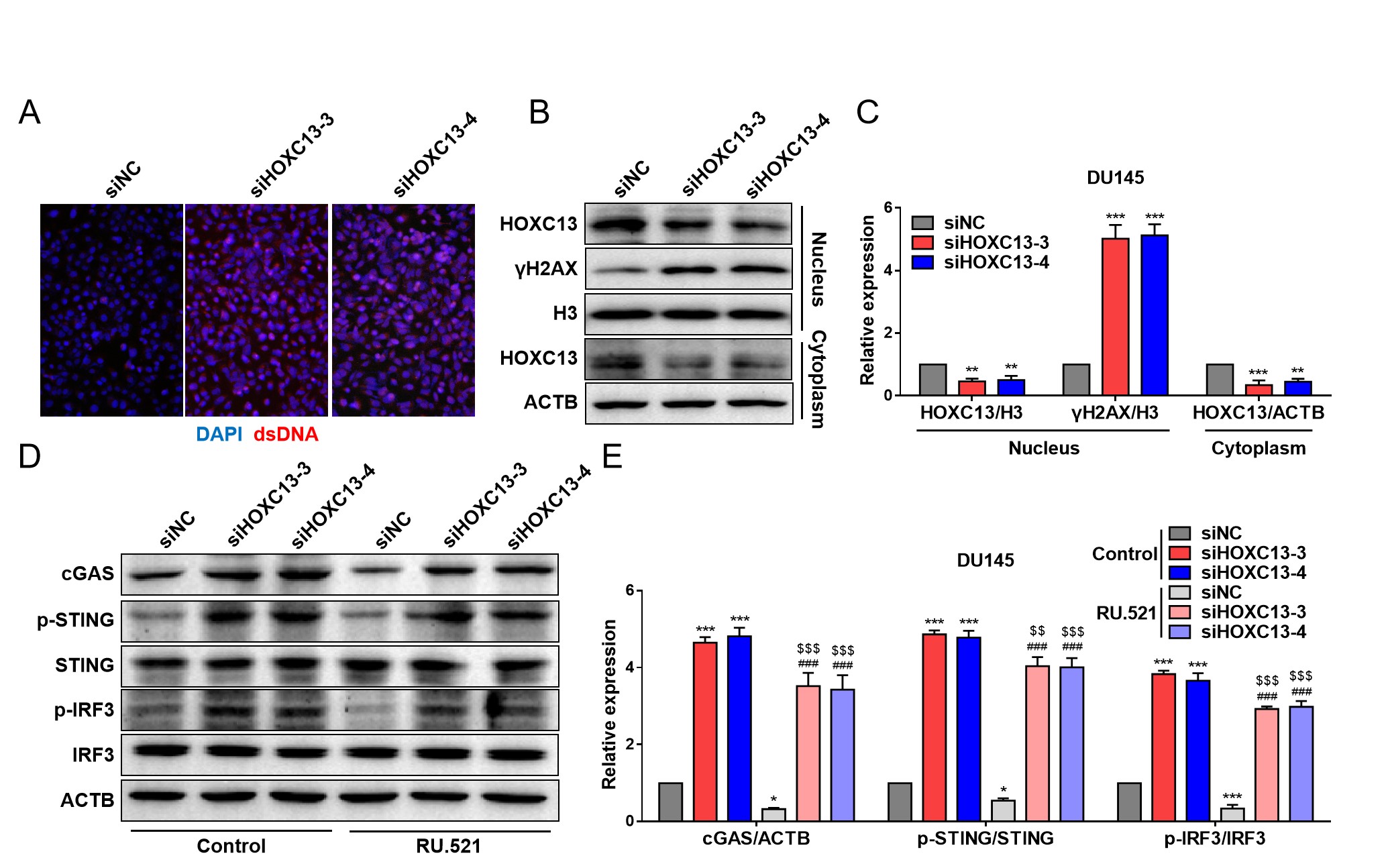

Supplement: Supplementary file 6 — Additional file 6: Figure S6. Effects of silencing HOXC13 on DNA damage-induced cGAS/STING/IRF3 pathway in DU145 cells. (A) Accumulation of dsDNA in cells. (B) Protein bands for nucleus and cytoplasm. (C) Expression statistics of HOXC13 and γH2AX proteins. (D) Protein bands for cytoplasm. (E) Expression statistics of cGAS, p-STING, STING, p-IRF3 and IRF3 proteins. (vs. siNC group: *P < 0.05, **P < 0.01, ***P < 0.001; vs. siHOXC13-3 group: ###P < 0.001; vs. siHOXC13-4 group: $$P < 0.01, $$$P < 0.001). [file 12967_2023_4743_MOESM6_ESM.jpg]
